# News and Coming Events

**Published:** 1908-07-25

**Authors:** 


### News and Coming Events.

The total of the Hospital Sunday collections to date exceeds £37,000.

Dr. J. Hill Abram, physician to the Liverpool Royal Infirmary and lecturer in the clinical school, has been elected to the chair of Therapeutics in Liverpool University.

The Earl of Denbigh, Chairman of the Royal National Orthopædic Hospital, has received a cheque for £100 from Lady Wantage as a donation towards the special appeal fund for rebuilding the hospital.

Professor Salvin-Moore, having resigned the directorship of the Liverpool Cancer Research Committee, will on September 30 vacate the Professorship of Experimental Cytology in the University of Liverpool.

On July 21 a letter of congratulation, printed on vellum, was sent to Mr. Thomas Bryant, past President of the College of Surgeons, by direction of the Council of the College, on his attaining the age of eighty years.

The Council of King's College have elected as Fellows of the College, amongst others, Professor Norman Dalton, M.D., Mr. A. Boyce Barrow, M.B., F.R.C.S., Professor Albert Carless, M.S., F.R.C.S., and Professor W. B. Bottomley, M.A.

Dr. Collingridge, medical officer of health for the City of London, draws attention in his annual report to a careless system of dispensing which has become common. The correct quantity of the drug prescribed is measured or weighed, and the bottle is then filled up with water, without any test being made to ascertain whether the bottle is of the proper size. A circular letter on the subject has been addressed to City chemists, many of whom now affix a special label to all bottles of medicine compounded by them certifying the accuracy of the contents.

During the hearing of a probate action on July 14, leave was asked for a medical witness to remain in Court, notwithstanding the recent order that in all contested cases witnesses shall remain out of Court until called. In acceding to the request Mr. Justice Bargrave Deane said he did not think the rule was intended to apply to medical witnesses. It is satisfactory to have this definite ruling on the point, though it is unthinkable that there was any intention of upsetting the long-established and necessary usage by the order referred to.

By the death of Sir John Banks, which took place on July 16, the medical profession in Ireland loses one of its most distinguished members. It appears that the exact date of his birth is unknown, but there is good ground for supposing the deceased physician to have been in his ninety-eighth year. Sir John Banks became M.D., Dublin, in 1843, and F.R.C.P.I. in the following year. He was President of the Irish College in 1869-71, and Regius Professor of Physic at Dublin for eighteen years. Sir John was also at the time of his death Physician-in-Ordinary to the King in Ireland, and held the corresponding post during the reign of her late Majesty. He was consulting physician to Sir Patrick Dun's, the City of Dublin, the Coombe, and the Royal Victoria Eye and Ear Hospitals. He was, moreover, a man of the most varied gifts and attainments, and there is probably no one now living whose experience covers so wide a knowledge as did his of the Viceregal Courts and society during Queen Victoria's reign.

This year's collection of the Australian Hospital Saturday Fund has beaten the best hitherto on record by nearly £1,100. The amount collected up to the departure of the last mail was £6,863 odd.

Arrangements have now been completed for two courses in school hygiene to be held next session under the direction of Professor H. R. Kenwood, assisted by Dr. H. Meredith Richards and other specialists. The first course will begin in October and will last until March. It will be held on Friday evenings at 7.15 and will be organised to meet the needs of school teachers. The second course will begin early in 1909 and will deal with school hygiene and medical inspection of scholars, and will be arranged to meet the needs of medical men.

The Congress of the Royal Institute of Public Health was formally opened by the Duke of Devonshire at Buxton on July 18. The President, in his address, dealt mainly with the question of tuberculosis in relation to milk supply, and pleaded for something like finality in the regulations imposed on farmers in the matter. Sir J. Crichton Browne, Dr. Bemrose, Professor Oliver, Major Ronald Ross, Sir Chas. Cameron, and others took part in the proceedings that day. On the resumption of the Congress on July 20 Sir James Crichton Browne, as President of the section of Preventive Medicine, delivered an address on "Parcimony in Nutrition."

The programme of business arranged for the forthcoming meeting of the British Medical Association at Sheffield includes a reception by the Lord Mayor on July 27 at 4.30 p.m.; on the 28th services at the Parish Church and at St. Marie's Roman Catholic Church, both at noon, and at 8.30 p.m. Mr. Simeon Snell's presidential address and the presentation of prizes in the hall of the University; on the 29th the address in medicine at 12.30 p.m., a reception at the works of Messrs. Jessop and Company at 2.30 p.m., a meeting of Convocation for conferring honorary degrees at 3 p.m., and a civic reception by the Lord Mayor, Master Cutler, and citizens of Sheffield in Weston Park at 8.30 p.m.; on the 30th is the address in surgery at 12.30 p.m.; and on the 31st the popular lecture at 8 p.m. and a reception by the President and local members of the Association. From July 28 to 31 inclusive the Food and Drugs Exhibition will be open at 9 a.m., and from July 29 to 31 the sectional meetings will be held from 10 a.m. to 1 p.m.

The annual meeting of the Poor Law Medical Officers' Association of England and Wales took place in Hull on July 15. Major Greenwood, M.D. (the Hon. Secretary), read a paper on "The Poor Law Medical Service," with special reference to the grievances of district medical officers. He said that whilst the importance of the service was constantly increasing, the remuneration was practically the same as it was sixty years ago. Extra fees amounted to little in the aggregate, and of late they had steadily diminished. The Poor Law service had been chiefly injured by anti-vaccination tactics of a covert character. Guardians made use of the public vaccinator to save the rates by paying him a smaller salary as a medical officer if he held that position. Resolutions were passed expressing the view that the system of remuneration urgently needs revision, and that better provision should be made for obtaining a skilled anæsthetist when needed, and condemning the practice said to exist of boards of guardians utilising emoluments of public vaccinators to supplement the inadequate remuneration of district medical officers.

The new Cottage Hospital at Sutton Coldfield, which has been erected at a cost of £2,500, was opened at the beginning of July.

---

The death is announced, at Eastbourne on July 20, of Alexander Charles Macrae, M.D., formerly Inspector-General of Hospitals in India, at an advanced age.

---

The Ashton-under-Lyne District Infirmary has received a cheque for £1,000 from Mr. Samuel Newton, J.P., to be applied in aid of the funds of the infirmary. The donor expresses the wish that the interest on the amount should go towards the endowment of a child's cot, to bear the name of himself and his wife.

---

The management of the Cardiff Infirmary have received £1,000 from "A Welsh Colliery Owner" towards the income fund. This donation is in response to Mr. John Corry's recent offer that if £5,000 a year is contributed to the income of the infirmary for the next two years he will add 25 per cent. to it.

---

By the will of the late Mrs. Waldo-Sibthorp, the Royal Hospital for Incurables receives £20,000, Charing Cross Hospital £10,000, and the Naval Hospital at Haslar £6,000. After other legacies and bequests have been paid, the residue of the estate is left on trust to the West End Hospital for Epilepsy, Welbeck Street, for the building and endowing of a new ward therein.

---

The new block at the Queen's Hospital, Birmingham, will, it is expected, be formally opened next October. The cost of the entire scheme, which includes improvements in the pathological department and the laundry, which were carried out a year or two ago, and the extension of the nurses' home, finished last year, has been £40,000. The new block will contain 60 beds, and can provide for 84 beds if required.

---

The Local Government Board has lately suggested that, as a means of relieving the pressure upon the accommodation for the sick poor of the metropolis, the Metropolitan Asylums Board should be empowered to provide accommodation for such sick or convalescent children as may be fit for removal, and suitable for reception into any buildings the managers may have available for the purpose. At an ordinary meeting of the latter board on July 18, the Chairman moved: "That the Local Government Board be informed that the managers are prepared to undertake the additional duties suggested by the Board, and to use the Southern Hospital for that purpose." It appears from the arguments with which this motion was supported that for two years this particular hospital has been vacant, though even in that state its upkeep costs from £1,500 to £2,000 a year; also that even in the fever epidemic last year it was not found necessary to open it, and that at the present time there are 931 fewer cases under treatment in the Asylums Board hospitals than was the case a year ago. In the opinion of the chairman there is no better use than that suggested to which this empty hospital can be put. The Vice-Chairman seconded the motion. After considerable discussion it was decided to take the opinions of the hospital managers upon the proposal, and the entire question was referred for consideration to the General Purposes Committee. It was further resolved to forward a copy of the letter to the metropolitan boards of guardians, asylum districts, and medical superintendents of infirmaries.

Queen Charlotte's Hospital has received a grant of 50 guineas from the Mercers' Company.

---

Mr. G. C. Mathison, M.B., B.S., has been appointed to the Sharpey Research Scholarship in Physiology at University College. The new physiological laboratories that are being built through the generous assistance of Dr. Ludwig Mond and Dr. Aders Plimmer are making rapid progress and will probably be ready for occupation early next year.

---

In answer to a question put by Sir W. Collins, the Premier, on July 20, announced that the Senate of the new Irish University will not be restricted from giving full recognition, should it think fit, to the work done in the (Irish) Royal College of Surgeons. He further hopes that if the working of the new system should prove injurious to the College the "Treasury may be found not indisposed to consider favourably an application for putting the College in a position to continue its work."

---

At a special general meeting recently held to consider the serious financial position of the Harrogate Infirmary, it was pointed out that the income of the infirmary is lower than that of any similar institution in the country. A resolution altering the rules with regard to the qualification of governors and moving that the number of beds available be reduced temporarily to such as the income of the institution warranted was submitted and seconded, but after discussion it was decided that the meeting be adjourned to some date in October, in order that the public might be made acquaintde with the position of affairs. It was stated that the great want of money was due to the enlargement of the hospital, which had cost £8,000, and entailed an extra staff, but that such enlargement was an absolute necessity for the efficient carrying out of the work of the hospital.

---

The Postmaster-General received on July 21 a deputation of members of Parliament and others on the question of the vaccination regulations now in force in regard to Post Office servants. The deputation was introduced by Sir W. Collins, M.P., who said his object was to suggest that the regulations in regard to the vaccination of Post Office employés might be revised. The Postmaster-General, in reply, said it is obvious that postmen are unusually liable to infection, and that no complaints have reached him either from their association or from individual members of the service. He supposed the deputation raised the question as one of policy, and would therefore not expect an immediate answer; he would inquire into the matter.

---

Lord Hugh Cecil's Infant Life Protection (No. 2) Bill has been under consideration by a Standing Committee of the House of Commons. A memorandum prefaced to the Bill states: "It is felony punishable by penal servitude for life to procure miscarriage. It is murder to kill a fully-born child. To destroy a child while it is being born . . . is no offence whatever. The object of this Bill is to put an end to this anomaly." The most important amendment carried is one dealing with the case of a medical student or midwife faced with an emergency demanding embryotomy. The following proviso has accordingly been added to the Bill as drafted: "Provided that a person shall not be guilty of an offence under this Act who by means employed in good faith for the preservation of the life of the mother of the child destroys the life of any such child during its birth."

